# GWAS of neuropsychiatric symptoms in Mild Cognitive Impairment and Alzheimer’s Disease/Related Disorders

**DOI:** 10.1002/alz.086866

**Published:** 2025-01-03

**Authors:** Aliza P. Wingo, Selina M. Vattathil, Thomas S. Wingo

**Affiliations:** ^1^ Emory University, Atlanta, GA USA; ^2^ Emory University School of Medicine, Atlanta, GA USA; ^3^ Department of Neurology, Emory University School of Medicine, Atlanta, GA USA; ^4^ Emory Goizueta Alzheimer’s Disease Research Center, Atlanta, GA USA

## Abstract

**Background:**

Approximately 85% of individuals living with MCI or ADRD experience one or more neuropsychiatric symptoms (NPS), referred to as ADRD‐NPS. They include depression, anxiety, irritability, apathy, agitation, delusions, hallucinations, and sleep disturbances. ADRD‐NPS are associated with greater functional impairment, higher caregiver burden, and earlier institutionalization. However, we have limited insight into mechanisms underlying ADRD‐NPS to develop effective treatments. Here, we conducted a GWAS of ADRD‐NPS to investigate genetic loci contributing to ADRD‐NPS.

**Method:**

We included only participants with MCI or ADRD in the GWAS of ADRD‐NPS. A total of 12,728 participants of European ancestry recruited by the U.S. Alzheimer’s Disease Research Centers were included. Among them, 23% had MCI and 77% had ADRD. ADRD‐NPS were assessed with the NPI‐Q. We conducted a GWAS for each of the ADRD‐NPS domains. Additionally, we performed a multivariate GWAS of all ADRD‐NPS domains simultaneously using a linear mixed model.

**Result:**

Both the GWAS of anxiety and the GWAS of delusion identified several genome‐wide significant SNPs (p < 5 × 10^‐8^) located in a 41 Kb region of chr19q13.32 after adjusting for sex, MCI/ADRD status, genotyping batch, and 10 PCs. Since this region overlaps the APOE gene and thus these signals may be related to severity of cognitive impairment (MCI or ADRD), we performed a stratified GWAS in MCI and ADRD separately. The stratified GWAS found that some of these signals remained genome‐wide significant in ADRD participants (n = 9766) for both anxiety and delusion, respectively. Furthermore, we found that APOE e4 allele was strongly associated with ADRD‐NPS in the domain of anxiety (p = 1.9 × 10^‐10^), hallucinations (p = 1.6 × 10^‐5^) and delusions (p = 4.0 × 10^‐12^) after adjusting for sex, MCI/ADRD status, genotyping batch, and 10 PCs. Lastly, the multivariate GWAS of ADRD‐NPS identified genome‐wide significant SNPs on chromosome 14 (KIF26A) and 19 (APOE) after adjusting for sex, MCI/ADRD status, genotyping batch, and 10 PCs.

**Conclusion:**

APOE e4 and genetic variants in chr19q13.32 are associated with anxiety and delusion experienced by individuals with MCI or ADRD. We plan to integrate the GWAS findings with human brain proteomic data to identify brain proteins contributing to ADRD‐NPS to nominate targets for therapeutic investigation.

